# Experiences of sharing results of community based serosurvey with participants in a district of Maharashtra, India

**DOI:** 10.1371/journal.pone.0271920

**Published:** 2022-08-04

**Authors:** Neha Salvi, Krishna Chaaithanya Itta, Abhishek Lachyan, Alvira Z. Hasan, Christine Prosperi, Muthusamy Santhosh Kumar, Jeromie Wesley Vivian Thangaraj, Ojas Kaduskar, Vaishali Bhatt, Gajanan N. Sapkal, Manoj Murhekar, Nivedita Gupta, Sanjay Mehendale, Kyla Hayford, William J. Moss, Sanjay Chauhan, Ragini Kulkarni

**Affiliations:** 1 Department of Health Research, Model Rural Health Research Unit, Dahanu, Maharashtra, India; 2 ICMR- National Institute for Research in Reproductive and Child Health, Mumbai, India; 3 International Vaccine Access Center, Department of International Health, Johns Hopkins Bloomberg School of Public Health, Baltimore, Maryland, United States of America; 4 ICMR-National Institute of Epidemiology, Chennai, India; 5 ICMR-National Institute of Virology, Pune, India; 6 Division of Epidemiology and Communicable Diseases, Indian Council of Medical Research, New Delhi, India; 7 PD Hinduja Hospital and Medical Research Centre, Mumbai, India; National Institute of Biologicals (Ministry of Health and Family Welfare, Government of India), INDIA

## Abstract

A growing number of organisations, including medical associations, recommend that research subjects should be given the option of being informed about the general outcome and results of the study. We recently completed a study involving nine serosurveys from 2018 to 2020 in five districts of India among three age groups (children 9 months to < 5 years; 5 to < 15 years of age, and women 15 to < 50 years of age before and after the measles and rubella (MR) vaccination campaigns). In Palghar district of Maharashtra all individuals in 30 selected clusters were enumerated, and 13 individuals per age group were randomly sampled. We established the procedures to return the results to the respondents for each stage of the survey. Of the 1,166 individuals selected for the measles and rubella serosurvey, 971 (83%) agreed to participate and were enrolled. Participants were informed that they will only be contacted if they test seronegative for measles and/or rubella antibodies. Overall, 140 individuals enrolled in the survey tested seronegative for IgG antibodies to measles and/or rubella viruses; were provided the reports and informed to seek medical advice. Upon follow up by phone, 10% (14) of the 140 participants reported to have been vaccinated. In this paper we discuss the procedures, experiences and considerations in returning results to participants in a community-based measles and rubella serosurvey. Although the lessons learned are specific to post measles-rubella vaccine campaign serosurvey in India, they might be helpful to those contemplating sharing results to participants of large scale survey settings.

## Introduction

The desire to respect the interests and preference of research participants by communicating results versus the responsibility to protect them from uncertain and poorly validated information has led to long-standing dilemma on revealing the research related results in biomedical research. Therefore, in most research studies, the researchers prefer to not share the results with the study participants. Investigators of community-based studies have been reported to engage with the participants extensively and then disconnected themselves from the participants, the situations described as “helicopter research” [1]. In contrast, practice of sharing results of a scientific study with participants is not only considered ethical but can also make study participants feel valued, help to build trust between researchers and the participants, and improve participation rates [2].

In view of the above, medical associations recommend that research subjects should be given the option of being informed about the general outcome and results of the study [1, 3]. In India, national guidelines for health research involving human participants recommend that efforts should be made to communicate the findings of the research study to the individuals and their communities [4]. The research team should also include plans to inform the study participants, including those in the control group, about the benefits they can avail and access after the study, in the research protocol [4]. Research sponsors and funding agencies also routinely require that applications for funding consistently address this issue [1]. Medical organizations have developed guiding principles for returning individual results to participants [5, 6]. These principles include an ethical responsibility to return a validated relevant result which is medically actionable, either directly to the participant or the physician(s) with primary responsibility to provide care to the individual on a case-to-case basis, and to document the communication and transfer of responsibility.

Serosurveys measure the prevalence of antibodies against an infectious pathogen in blood and can provide a direct measure of population immunity [7]. Serosurveys most commonly measure IgG antibodies, a marker of past exposure to a pathogen or receipt of a vaccine. Given that IgG antibody assays typically measure past exposure instead of active infection, the potential actions to be taken by participants differ relative to research studies using diagnostic tests (e.g., vaccination rather than medical care for an acute infection). The exceptions are chronic viral infections, such as HIV, for which detection of IgG antibodies indicates infection.

We conducted a serological survey before and after a measles and rubella (MR) vaccination campaign in Palghar District of Maharashtra state in India to estimate measles and rubella seroprevalence. In our serosurvey, results were returned to individuals found to be seronegative to measles or rubella IgG antibodies after the MR vaccination to make them aware of this and enable them to consider the opportunity to be vaccinated. In this paper, we have discussed the procedures, experiences and other considerations in returning results to participants in a community-based measles and rubella serosurvey. Although the lessons learned were specific to a post MR campaign serosurvey in India, they might be helpful to those contemplating sharing results to participants of large scale survey settings.

## Methods

### Survey method

A multi-stage sampling design was used to conduct nine serosurveys from 2018 to 2020 in five districts of India among three age groups (children 9 months to < 5 years; 5 to < 15 years of age, and women 15 to < 50 years of age [post-campaign only]) conducted before and after the MR vaccination campaign. The overall objective of the study was to estimate age-specific measles and rubella seroprevalence and to evaluate the impact of the MR campaign. Here we focus on the survey conducted in Palghar District, Maharashtra conducted from April to June 2019, about 3 months after the MR campaign. The detailed study methods have been reported elsewhere [8]. Briefly, 30 villages or wards were first selected from the district based on the 2011 census, then one census enumeration block or cluster was randomly selected from each village. In each cluster, all individuals were enumerated, and 13 individuals were randomly selected per age group. Prior to enrollment, informed consent was obtained from all individuals. This included explanation about the IgG test and what it means to be positive or negative during the informed consent process. After obtaining consent, survey staff collected information on socio-demographic and vaccination history data from parents or caregivers of the selected child. If the child had not received the vaccine, the parents were encouraged to seek vaccination. A venous blood sample was collected and sent to the Indian Council of Medical Research (ICMR)-National Institute of Virology (NIV) Pune, to test for IgG antibodies against measles and rubella viruses.

The surveys were led by the ICMR-National Institute of Epidemiology, Chennai, and locally implemented by researchers at the Model Rural Health Research Unit (MRHRU) in Palghar district and the ICMR-National Institute for Research in Reproductive and Child Health (ICMR-NIRRCH).

#### Study setting

Palghar District, Maharashtra has been carved out of Thane district of Maharashtra in August 2014. As per the 2011 census, this district had a population of 29,90,116, with 48% living in urban areas. A recent survey has reported that almost half of the women of reproductive age have attained 10 years or more of education [9].

#### Returning results to participants

**Fig 1:**
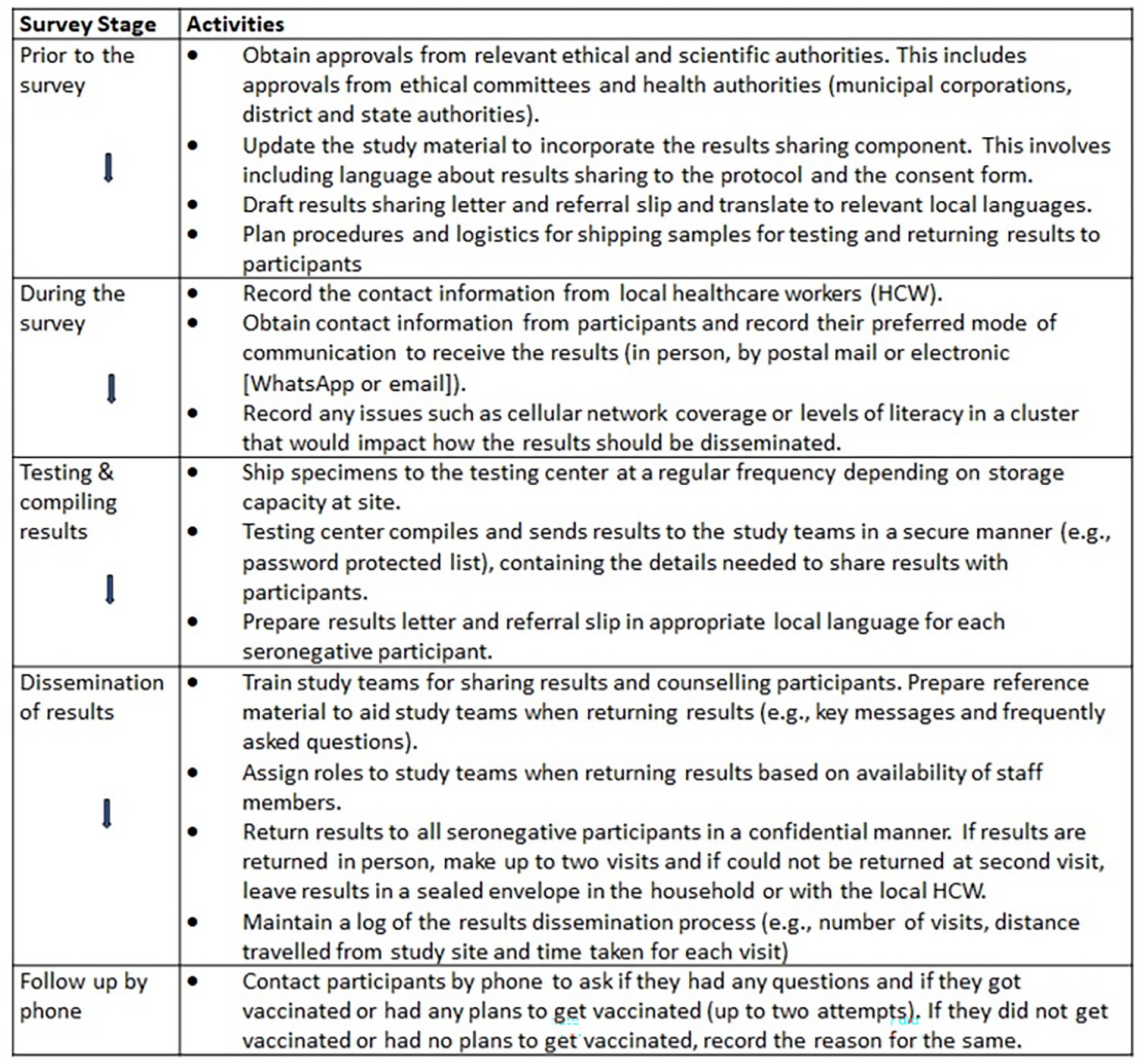
Overview of survey stage and activities involved in returning results to participants.

Results of the pre-campaign survey were not returned to participants since the MR vaccination campaign was scheduled shortly after the survey, and all children up to the age of 15 years were encouraged to get vaccinated in the campaign. In the post-MR campaign survey the study team, with recommendation of Institutional Ethics Committee of ICMR-NIRRCH, returned results to participants who tested seronegative to measles or rubella viruses owing to their vulnerable status. The consent forms were modified to explain that individuals who test seronegative for measles and/or rubella IgG antibodies will be informed of their test results and be advised to seek vaccination. Those who test positive for measles and rubella IgG antibodies will not receive results and should presume that their reports suggest seropositivity. The procedures were developed for returning results for each stage of the survey (Fig 1).

#### Prior to the survey

Incorporating the results sharing component into the study involved updating consent forms, obtaining approvals from relevant authorities, and drafting individual results letters. The key features of the participant’s result letter (S1 Appendix) included an explanation of the survey, testing site, the individual’s test result, a referral slip (S1 Appendix) for the nearest health facility to discuss the result with medical officer and contact information of the study team. The test result was provided with the caveat that the antibody test used was for research purposes to learn more about the population and should not be considered as individual clinical results. The letter advised pregnant women to wait until after delivery to receive the MR vaccine and non-pregnant women to avoid pregnancy for at least three months following vaccination. Standard Operative Procedures [SOP] were developed for shipping samples for testing on a regular basis and for how the results will be returned to the participants. Additional approvals were obtained from district health officials to permit the MR vaccine to be administered to individuals above two years of age on the routine public health system platform in the district.

#### During the survey

At the beginning of the survey in each cluster, telephone numbers were obtained from local health workers (HCW) to support study teams during the survey and help with returning results. Contact information and their preferred mode of receiving results was obtained from each participant in a secure manner. Participants were informed that they will only be contacted if they test seronegative for measles and/or rubella antibodies about 1.5–2 months after the survey. However, all the participants were provided with contact details of the study team and encouraged to contact if they wished to know about their results. The study teams also recorded issues such as cellular network coverage and levels of illiteracy in a cluster that would be helpful in planning the best mode for results sharing. Generally, in rural clusters a personal visit was the default mode to return results while in urban clusters other options such as returning results by postal mail or electronically (WhatsApp or email) were offered.

#### Testing of samples and compiling results

After completing the survey, blood samples were shipped to the testing center. Following testing, a list of seronegative individuals was prepared and provided to the study teams. Using this information, the study team prepared a result letter and a referral slip for each seronegative individual in the local language.

#### Dissemination of the results

The study team coordinators and staff, fluent in the local language of the participants, were trained on how to share results with participants, including counselling participants with seronegative results and advising further course of action. As a part of the training, investigators provided reference material to aid the study team when returning results. This included a list of potential reasons why a participant tested seronegative and key messages the team should communicate with the results, described at a level understandable to the community (S2 Appendix). Initially the activity was carried out by two teams in the first 18 clusters, each consisting of a study coordinator and one or two team members accompanied by a HCW. Once the community survey was completed, only one team was retained for returning results in the remaining 12 clusters. The information was shared in a confidential manner with all seronegative participants or parents of child participants. HCWs helped field teams only to identify the households of participants but results were not shared with the HCWs to ensure confidentiality. The responses and questions from the participants regarding the results were noted and answered. The participants were encouraged to visit their local healthcare facility to discuss the results with the medical officer and to receive the MR vaccine if they wished so. A maximum of two visits were planned to return results in person to the participants. If results could not be returned after two visits, the results letter was to be placed in a sealed envelope addressed to the participant and left with their relatives or the local HCW. A record for the number of clusters completed in one day, the total duration of each visit, the time spent in each cluster and the distance travelled to complete each visit was maintained.

#### Follow up by phone

Up to two attempts were made to contact the participants by phone about 15 days to one month after sharing results. At the time of follow up, participants were asked if they had any questions about the results returned to them and if they had taken any action after receiving the results such as receiving the MR vaccine.

#### Ethical approval

Ethical approval for this study was obtained from Ethics Committee of ICMR- National Institute for Research in Reproductive and Child Health (Ref: D/ICEC/Sci-40/41/2019).

## Results

Of the 1,166 individuals selected for the measles and rubella serosurvey, 971 (83%) agreed to participate and were enrolled. On average the measles and rubella antibody results were compiled and shared two months after the specimens were collected (mean 62 days; range 47–84). Overall, 140 (14.4%) individuals enrolled in the survey tested seronegative for IgG antibodies to measles and/or rubella viruses. The average number of seronegative individuals per cluster was five (range 1–11).

All the enrolled participants requested receiving the result in person when asked during the survey, including urban participants.

We were able to return the results to all the participants who tested seronegative for antibodies to measles and/or rubella viruses. Results were communicated during the first attempt for all participants except for five (residing in three clusters) who required two visits. Only one participant out of the five who required second visit could not be reached and hence the result was left in sealed envelope. Eighty-seven percent (122/140) of participants or their caregivers received the results directly from the study team. For the remaining 18 participants, the results letter was left in a sealed envelope with either an elder family member (9.2%, n = 13) or with a local health worker (2.8%, n = 4) on insistence of study participant / parent of child participant even though the participant was available in person. One (0.71%) participant could not be contacted after two attempts and the result was left in a sealed envelope with a local health worker.

The mean distance between the study site and the cluster was 162 km (minimum 40, maximum 232 Kms.). A total of 22 visits were undertaken to complete dissemination of the results to participants in 30 clusters. The mean time taken to complete one visit (time taken to travel from site to and from the cluster and time spent in the cluster) was approximately 7 hours (minimum 4 hours, maximum 11 hours) and the mean time taken to disseminate result in one cluster was 3 hours and 38 minutes (minimum one hour, maximum 7 hours).

Only two participants inquired why vaccination was advised upon receipt of results. Most of the others agreed to see their medical officer to discuss the results and get vaccinated if advised. Three pregnant participants were advised vaccination after delivery of the baby as per the recommendation of their doctor or local health facility.

Upon follow up by phone, 10% (14) of the 140 participants reported to have been vaccinated, 11.4% (16) were not ready to get vaccinated, 69.2% (97) planned to get vaccinated at a later date, 2.4% (3) were pregnant, medical officer advised that no vaccination was necessary in 0.7% (1) and we were unable to contact either the participant or their health workers in 6.4% (9) participants. Of those who were not ready to get vaccinated, some participants mentioned that this was due to rumours that vaccination will lead to infertility, but others did not provide a reason for not getting vaccinated.

## Discussion

**Fig 2:**
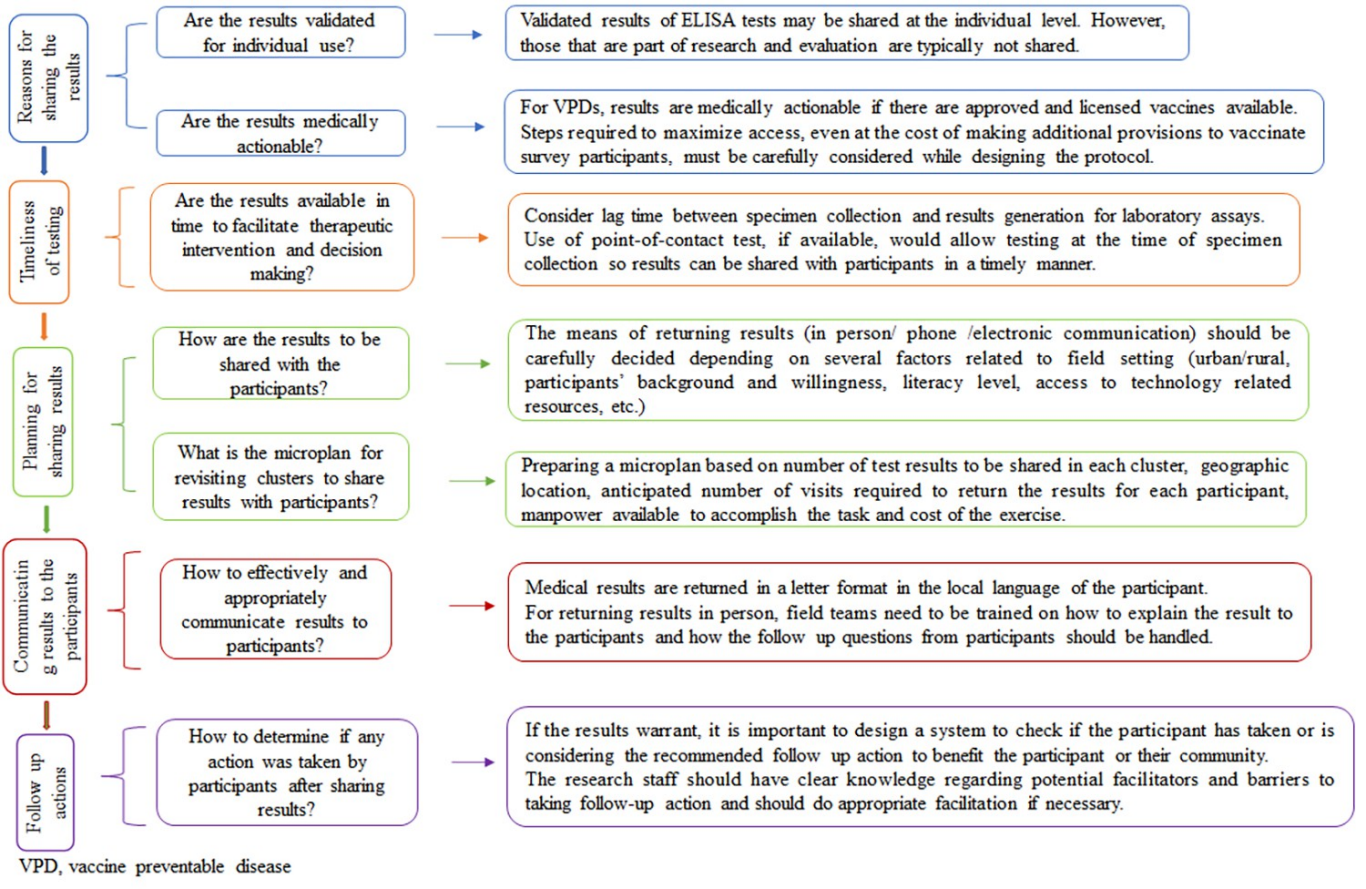
Decision flowchart to be used while returning results to participate of large scale serosurveys.

Returning results to participants of a research study can be challenging and this study explored the challenges and the measures taken to successfully return the results. In this household serosurvey, results were successfully returned to all 140 participants who tested seronegative to measles and/or rubella with only five requiring a revisit. Although we were successful at returning results to participants, there were many important considerations we faced related to whether results should be shared, the planning steps for returning results, how to effectively communicate results with participants, and what additional follow up with participants may be done to determine if any action was taken. We present these operational perspectives for returning results to participants with examples from our experiences in this serosurvey (Fig 2).

In terms of whether results can be returned to participants, one must consider whether assays are validated for individual use and whether results are medically actionable. The enzyme immunoassay kits used for Measles and Rubella IgG detection in MR serosurveys were Conformite Europeenne (CE) approved and designed for use as IVD (In vitro Diagnostic Medical Device). Following the MR campaign, those who tested seronegative for antibodies were potentially susceptible to measles and/or rubella. Measles is a highly infectious and outbreak-prone disease that kills many children in India and can also lead to life-long disabilities, while rubella causes congenital rubella syndrome (CRS) in newborns of women infected in early pregnancy. Since both measles and rubella have approved vaccines available to prevent disease, the serosurvey results are medically actionable. Therefore, the local ethics committees opined and advised that it would be ethical to return results to participants so that they are aware of their serostatus and have the option to take action given that those found to be seronegative after the MR campaign could be at risk of infection and disease.

Despite availability of an approved MR vaccine, the public health system in India routinely only provides the MR vaccine to children under 2 years. For those above 2 years, the options for receiving the vaccine are limited to private health providers who are typically harder to access due to financial barriers. To ensure that participants who were potentially susceptible to measles and/or rubella are not denied MR vaccine, special permission was obtained from district health authorities to provide MR vaccine to older children and adult women in the public sector. Thus, it is important to note that decisions related to returning results depend on various factors related to the test used, whether there are medical actions individuals can take to prevent or treat the target disease, and what is ethical in each setting. For diseases that are not vaccine preventable, it may not be possible for participants to take any action once they know of their status. However, even if a vaccine is available, there are other factors to consider such as age restrictions within the routine immunization policies and access barriers.

When planning to return results, the process and timelines for testing need to be planned in advance. In this study, since testing was conducted in a central laboratory, the results were not immediately available. The specimens needed to be shipped from the field site to the testing centre at a regular frequency. The time taken to collect, ship, test and compile results needed to be evaluated prior to starting the survey so that accurate turnaround times could be communicated to field teams and the participants. Additionally, this time lag affected staffing needs as staff needed to be retained after the survey to return results. Thus, if assays need to be performed at a laboratory, there is typically a lag between specimen collection and results generation. Other testing alternatives include point-of-contact tests (POCTs) or rapid diagnostic tests which, if available, could potentially allow results to be shared with participants immediately after testing. Rapid diagnostic tests are commonly used in other surveys are typically for antigen detection, as opposed to antibody which serosurveys detect, very commonly used in malaria, Chlamydia and more recently in SARS-CoV-2 POCTs to rapidly generate results [10]. A study has explored the potential value of rapid measles antibody (specifically IgM) testing in global measles surveillance [11]. Another study, has explored the value of rapid antibody testing for HIV in community and outreach settings [12]. Availability of rapid tests would increase the amount of time the staff spend with the participant on the day of enrolment, but removes the subsequent steps of compiling results and separate visits to share results. Rapid testing also requires investigators to develop study materials for returning results and train staff on how to communicate results prior to the survey starting, rather than during or after survey completion, so the staff are prepared to immediately share results and provide medical treatment or referral.

Returning results from a serosurvey requires coordination between the study team, the technical team conducting the test, the investigators and the local health workers and officials. This exercise requires additional manpower and transportation which involves additional cost. In this serosurvey, participants and parents were informed that the results will be shared with them if they test seronegative, and their preference to receive the result and the mode by which they wish to receive the results was noted during enrollment. Though the urban participants could opt for any mode (in person, phone, electronic), all participants across urban and rural settings chose to receive the results in person. Returning results in person was challenging as it required additional resources in terms of staff, time, and transportation compared to other modes such as via the phone or email. However, sharing results in person provided the opportunity to interact with participants to help explain their test results, answer their questions about the test results, describe the importance of vaccination and explain the referral process for vaccination. The ideal mode of returning results will depend on the setting, and there are other important considerations to alternative modes such as availability of cell phones and phone network, internet connectivity, proper addresses, and literacy level of participants [13–17]. There are very few studies in India regarding preferred mode of receiving research results [18]. Knowing participant’s preference was helpful in planning results sharing procedures in terms of training staff, preparing appropriate format for results, planning travel and estimating the cost for the entire exercise. In preparing a microplan for returning results we considered the geographical location of each cluster relative to the research site, number of participants in the cluster with whom the results had to be shared, the proximity of the clusters with each other to reduce the number of trips required, and availability of the participants with regards to when people leave for and return from work and seasonal migration. In some situations, unavailability lead to scheduling a revisit or not being able to share the result directly with the participant.

When returning results to participants in a research study, study teams must ensure the meaning of results are communicated effectively. Typically, medical results are returned in a letter format in the local language of the participant. In this serosurvey, the content of the results letter was carefully developed to clearly describe what being seronegative to measles or rubella viruses means to differentiate it from testing negative for the disease. If participants were illiterate, field teams explained the result verbally in a confidential manner. The list of potential anticipated FAQs developed served as a helpful reference for field teams when communicating with participants. Thus, the content of the results letter, training field teams on how to explain the result to participants (if needed) and how to handle follow up questions from participants is important when returning results to participants in person.

After results are returned, investigators may wish to determine if any action was taken by participants after learning of their results. If so, it is important to consider what additional steps may be needed to complete this and what information would be of interest to collect beyond whether or not the primary action was taken. In this study, we were interested to learn if participants got vaccinated following receipt of results so we followed them up by phone. While most participants could be reached (93%), only 10% confirmed they were vaccinated with the MR vaccine. The remaining did not get vaccinated because they were “not ready” or planned to get vaccinated later, citing reasons such as pregnancy, hesitancy and unavailability of vaccine at the local health facility. Future studies could consider a separate qualitative follow up study to understand the motivators and drivers of those who took action and barriers for those who did not. Future studies could also consider ways to use the results sharing as an opportunity to engage with the local health workers to increase outreach and increase vaccination uptake in their communities.

In our experience, community members were more motivated to participate and had greater trust in the serosurvey knowing that the results will be returned to them. Anecdotally, staff encountered greater hesitancy and refusal during the pre-campaign serosurvey, when results were not shared, relative to the post-campaign. This is consistent with findings from other studies evaluating perspectives on sharing results. Long et al carried out a study involving 414 health researchers to understand their experiences, perceptions and barriers to sharing study results [2]. Advantages of sharing results are benefits to research or science due to increased participation, closure for study participants, participants feel valued and building trust between researchers and participants leading to long-term public engagement in research. A recent systematic review of returning individual research results from genomic research also demonstrated that research participants were greatly interested in receiving results and also a general willingness from investigators to provide such results [19]. When the researchers were asked about challenges and barriers to sharing results, they cited ethical concerns, financial, logistical, systems related, methodological and skill-related barriers [2]. Some of the ethical concerns cited are related to the validity of the results, how the results may be understood by the participants, whether the results will lead to confusion or cause emotional harm to the participant [2, 4, 20, 21]. All of these were considerations we addressed in planning and implementing results sharing for our serosurvey, as described above.

The procedures and experiences when returning results to participants described here are specific to this serosurvey and are influenced by the survey design, antigens targeted, assay used, and the study setting. Although these may not be directly generalizable to other surveys and research studies, the key considerations would apply to other contexts. (Fig 1) The results sharing procedures and implementation will need to be revised for other serosurvey contexts. For diseases such as SARS-CoV-2, there may be greater individual-level desire from the participants to receive their antibody results. However, given the pandemic context, additional in-person interactions to share results may put both staff and participants at risk of transmission. Alternative modes of sharing results, such as phone, text, or email, may be preferred, but may result in further complications such as inability to ensure the participant receives the results and has an opportunity to ask questions.

## Conclusion

This study contributes to literature on experiences and procedures when returning individual results to participants in person in a serosurvey. A point-of-contact assay may reduce the time and cost involved in sharing of results during serosurveys. Our experience with sharing results suggests that if the plan to share the results is included from the beginning in the protocol and if procedures are put in place including budget for the activity, sharing of results becomes a seamless exercise which is appreciated well by participants. While the experiences are specific to settings in India, considerations outlined from our experiences maybe generalizable to other serosurveys targeting other antigens.

## Supporting information

S1 Appendix Participant results letter and referral slip. (PDF)

S2 Appendix Key messages and frequently asked questions for communicating seronegative results to participants or parents / legal guardians receiving results. (PDF)
