# Supplementary material for: Experiences of sharing results of community based serosurvey with participants in a district of Maharashtra, India
Source: PLoS One. 2022 Aug 4;17(8):e0271920. doi: 10.1371/journal.pone.0271920 (PMC9352079; doi:10.1371/journal.pone.0271920)
Supplement: S1 Appendix — (PDF) [file pone.0271920.s001.pdf]

SUPPLEMENTARY APPENDIX 1

PARTICIPANT RESULTS LETTER
AND REFERRAL SLIP

Name:__________________________Age:_______Participant ID:_____________HH ID: ____

Impact of Measles Rubella Vaccination campaign on population immunity in India (IMRVI study)

Dear IMRVI study participant / participant's parent or guardian,

In________*2019* you / your child participated in a study to find out the level of protection against measles and rubella among people in your area and other parts of India, called the Impact of measles rubella (MR) vaccination campaign on population immunity in India (IMRVI study). Researchers from National Institute of Research in Reproductive Health and Model Rural Health Research Unit, Dahanu conducted the study.

During the study we collected a blood sample from you / your child to test for the presence of antibodies to measles and rubella viruses that protect against these diseases. The test was performed at the National Institute of Virology, Pune. Measles is a highly infectious and outbreak-prone disease that kills many children in India. Apart from death, measles can also lead to life-long disabilities, including blindness, brain damage and deafness.Rubella can cause birth defects in the newborn children of infected mothers.

You are receiving this letter because you / your child tested <u>negative</u> for the presence of antibodies to at least one of the viruses (see below):

- Measles antibodies: __________________
- Rubella antibodies: __________________

This means that it is likely you/your child are not protected against _____________________. There is a chance this test result may be incorrect and you / your child may be protected against ____________________as this test is only used to learn more about the population, not to give an individual their health result. Either way, we recommend vaccination to protect you / your child. You may use the referral slip below to access MR vaccination at your local health facility.
For adult women: If you are currently pregnant you should <u>not</u> be vaccinated. If you are not pregnant and get vaccinated <u>avoid pregnancy for 3 months after vaccination</u>.

If you wish to find out more about the meaning of these results or the study, you can contact the IMRVI study team on Phone no. xxxxxxxxxx

----

Referral slip

We are referring ________________________ aged _______years to your facility as his/her test result indicate that he/she is not immune against <specify disease>. We have attached a letter from DHO/CS/MOH VVMC regarding facilitating vaccination for them along with this referral slip.
Kindly provide MR vaccination at your facility. Kindly confirm that the woman is not pregnant as MR vaccine is contraindicated in pregnancy. Also, please advise the woman to avoid pregnancy for 3 months after vaccination).

Thanks & Regards,

Name & Signature of the IMRVI study staff: Date:

Name & Signature of the participant / parent or legal guardian receiving referral slip: Date:
