# Supplementary material for: Experiences of sharing results of community based serosurvey with participants in a district of Maharashtra, India
Source: PLoS One. 2022 Aug 4;17(8):e0271920. doi: 10.1371/journal.pone.0271920 (PMC9352079; doi:10.1371/journal.pone.0271920)
Supplement: S2 Appendix — (PDF) [file pone.0271920.s002.pdf]

**SUPPLEMENTARY APPENDIX 2**

**Key messages and frequently asked questions for communicating seronegative results to participants or parents / legal guardians receiving results**

When sharing the seronegative results, the minimum messages should include:

1. Test result indicates you are not protected against measles / rubella
    - Rubella is a rash illness that can cause birth defects and abortion in pregnancy
    - Measles is a rash illness that can cause death or life-long disabilities, including blindness, brain damage, and deafness in rare cases
2. Recommend getting vaccinated to protect yourself / your child.
3. No test is perfect and there's a small chance it is incorrect.
4. Can consult the medical officer or your doctor from *Site Name* if you have more questions.
5. Staff will inform the woman that if she is currently pregnant she should not be vaccinated until after delivery. If the woman is not pregnant she will be informed to avoid pregnancy for at least one month after vaccination (site recommendation may be longer). Do not probe about pregnancy status as it may be a sensitive topic.

**Frequently asked questions**

Why was my test seronegative if I have already been vaccinated?

- Small number of people who are vaccinated do not develop protection
- Rarely other conditions may influence the chance someone develops protection after vaccinations such as illnesses or cancer treatments causing immunosuppression.
- Test is used for research purposes only.
- No test is perfect and there's a small chance it is incorrect.

Do I need to be vaccinated again?

- Even if you were previously vaccinated, we advise you be vaccinated again to ensure you are protected against measles and rubella.

Can I be retested?

- Speak with your health provider for more information regarding retesting but retesting is generally not recommended.
